# Triple-Negative Male Breast Cancer Presenting as Cutaneous Metastasis: A Diagnostic Rarity

**DOI:** 10.1155/crom/4393243

**Published:** 2025-11-20

**Authors:** Saran Lal Ajai Mokan Dasan, Neelanjana Pandey, Mikhail Sukhoroslov, Donald Rudikoff, Naqash Mazhar

**Affiliations:** Department of Internal Medicine, BronxCare Health System, Bronx, New York, USA

**Keywords:** cutaneous metastasis, male breast cancer, secondary skin tumor

## Abstract

Cancer of the male breast is a rare disease. It comprises less than 1% of all breast carcinomas and less than 1.5% of all malignant tumors in men. Male breast cancer presenting as skin metastasis is exceptionally uncommon. A review of the medical literature identified only a handful of such cases. We present a malignant axillary skin tumor with multiple visceral metastases originating from a breast primary. Although often grouped together, recent evidence indicates that male breast cancer is a distinct tumor on both genetic and molecular grounds when compared to the significantly more prevalent female breast cancer. Since new details and treatment strategies are emerging for male breast cancer, we wish to highlight a rather unusual presentation of this often-overlooked cancer. Skin metastasis is generally detected at the terminal stage of the malignancy. In our patient, however, the skin metastasis initiated the diagnostic workup.

## 1. Introduction

Male breast cancer (MBC) is a rare condition, representing less than 1% of all breast cancer cases; however, its incidence has increased globally. According to 2020 American Cancer Society statistics, 2670 men were diagnosed with breast cancer, resulting in 500 deaths [1]. Identified risk factors are advanced age, obesity, testicular diseases, and tumors, as well as germline mutations in BRCA2. Carriers of the BRCA2 gene mutation have an approximately 80-fold increased risk compared to the general population [2]. On average, men are diagnosed with breast cancer at an older age (67 years), compared to 62 years for women [3]. MBC is most often classified histologically as Grade 2, typically hormone receptor–positive and Human Epidermal Growth Factor Receptor 2 (HER2)–negative. In situ and invasive papillary carcinomas are commonly observed [4]. Reporting and staging protocols for MBC are similar to those for female breast cancer. Metastatic lesions involving the male breast can occur and must be distinguished from primary breast carcinomas. Historically, MBC was considered analogous to the typical estrogen receptor (ER)–positive, postmenopausal female breast cancer. However, recent research and clinical trials have identified significant differences between MBC and its female counterpart regarding age at presentation, clinical characteristics, tumor origin, histopathological features, and patient outcomes [5]. Despite a later age of onset and more advanced disease at diagnosis, MBS patients have better survival than female breast cancer patients [6]. In anticipation of forthcoming research findings, ASCO has issued a guideline outlining recommendations for the management of MBC. Review of the literature in PUBMED, SCOPUS, and EMBASE with search terms MBC with skin metastasis revealed only four studies in the aforementioned pattern, signifying the rarity of this entity.

## 2. Case Details

A 77-year-old Hispanic male with a medical history of hypertension, hyperlipidemia, prior myocardial infarction, benign prostatic hyperplasia, and dementia was transported to the hospital by emergency services for evaluation of an acute onset nausea and vomiting that began after breakfast. He is normally cared for by his daughter, owing to his advanced dementia. She reported that he had been feeling dizzy but had not lost consciousness.

The patient demonstrated limited awareness of his medical condition and was unable to offer any details regarding his current or previous medical history. According to the patient's daughter, he was currently undergoing evaluation at a different hospital for a suspected primary skin cancer located on the chest wall and had been scheduled for a CT scan to assess the possibility of bone metastases.

**Figure 1:**
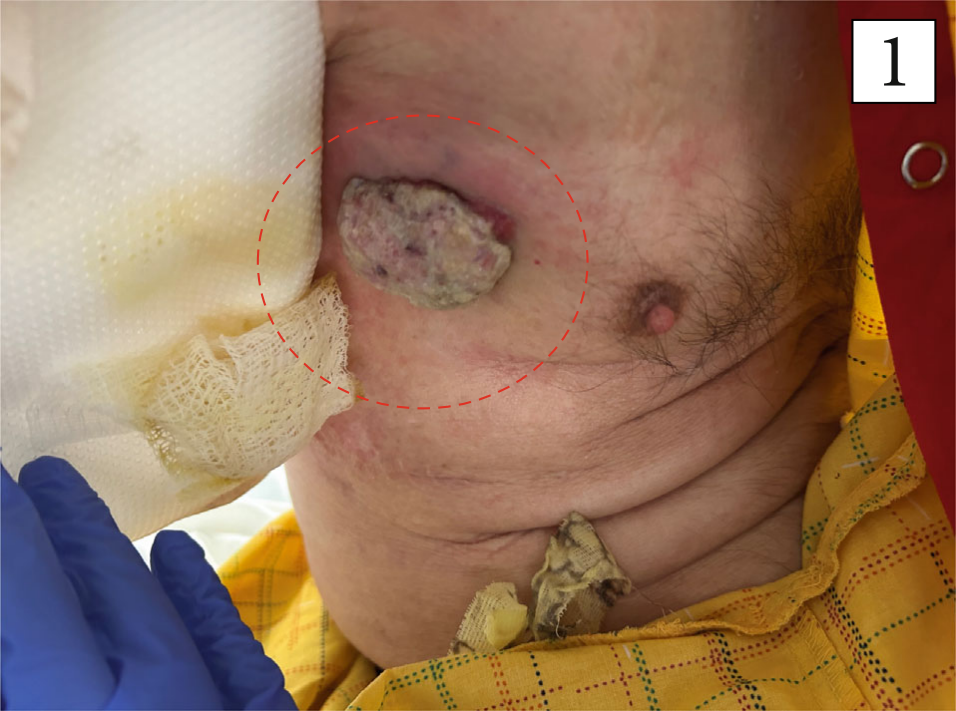
Clinical photograph demonstrating a 10 × 10 cm fungating mass on the right lateral chest wall with central necrosis.

**Figure 2:**
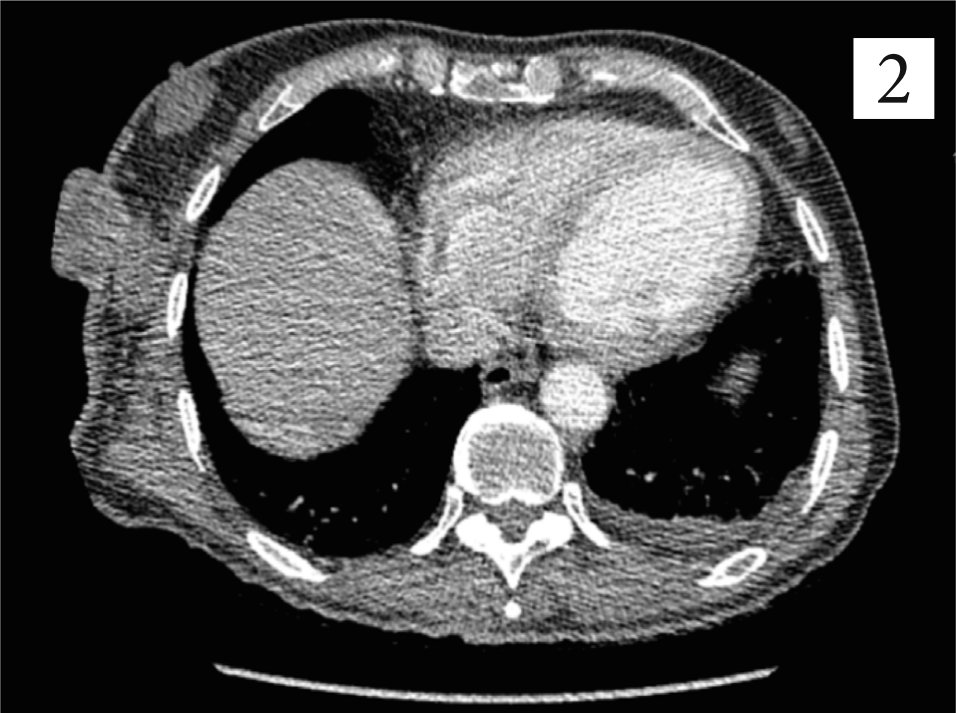
Axial CT scan of the chest illustrating an exophytic right chest wall mass with soft tissue extension.

On physical examination, a 10 × 10 cm fungating skin mass was noted on the right upper chest, with associated erythema and mild tenderness on palpation (Figures 1 and 2). Additionally, the right axillary lymph nodes were firm and markedly enlarged, and a superficial scar was observed on the right flank.

**Figure 3:**
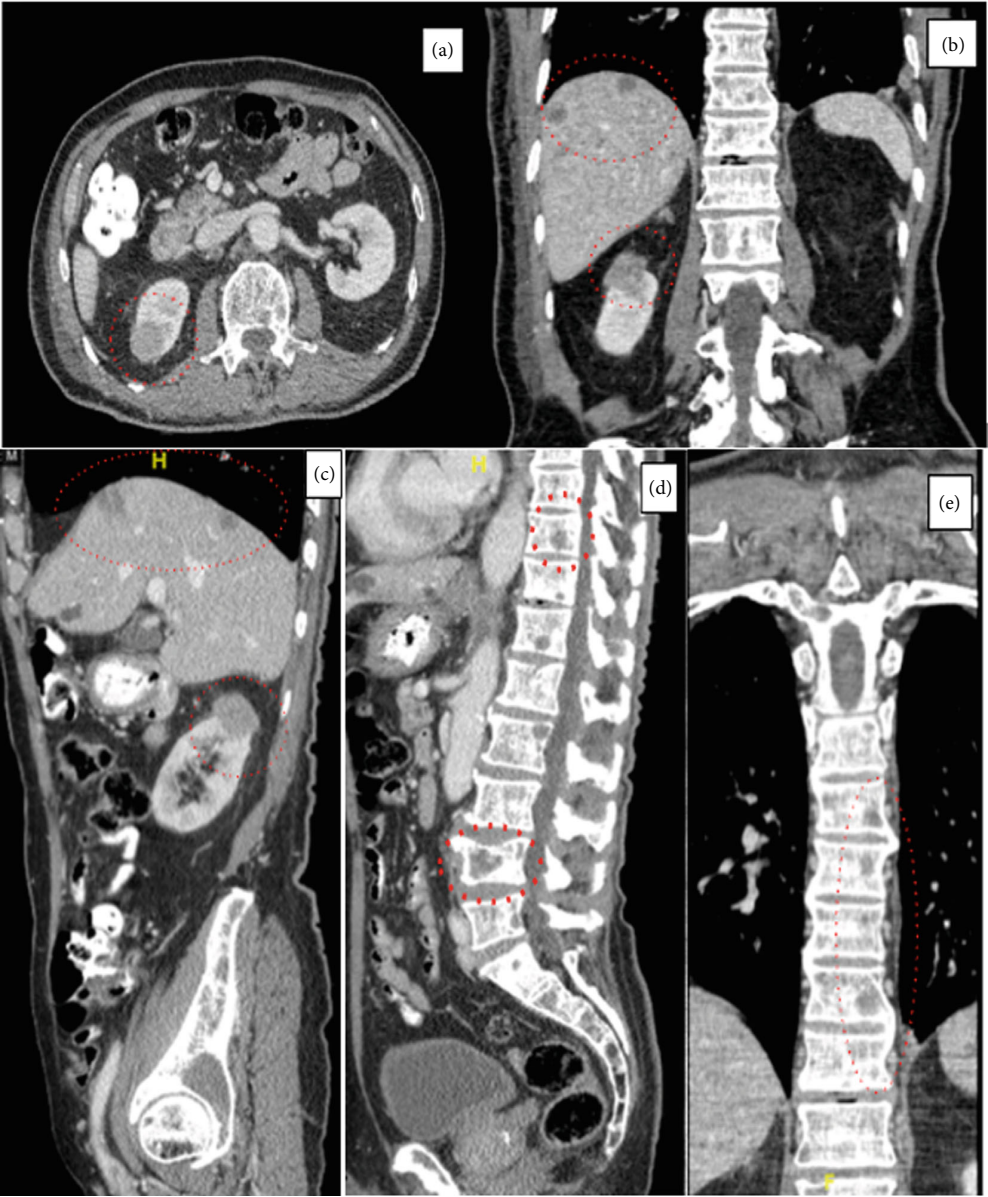
Computed tomography imaging demonstrating widespread metastatic disease. (a) Axial CT abdomen showing right renal metastasis. (b) Coronal CT abdomen revealing multiple liver and right adrenal metastases. (c) Sagittal CT abdomen with combined renal and hepatic involvement. (d) Sagittal CT spine showing extensive lumbar vertebral metastases. (e) Coronal CT spine with multiple skeletal metastases.

**Figure 4:**
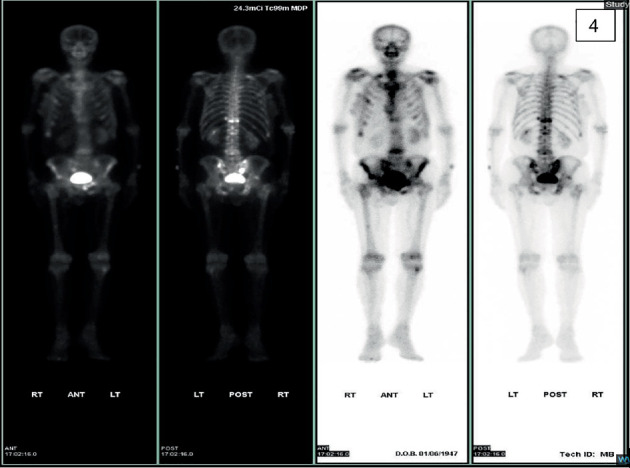
Whole-body bone scintigraphy demonstrating multiple foci of increased tracer uptake involving both axial and appendicular skeleton, consistent with widespread skeletal metastases.

Laboratory examination showed an elevated alkaline phosphatase and Aspartate aminotransferase. Imaging for acute abdominal symptoms showed extensive metastases in the lungs, liver, kidney, and adrenal gland (Figures 3 and 4). The patient was admitted for further monitoring and evaluation of the occult primary.

Records obtained from the prior hospitalization revealed two biopsies done for the skin lesion. Superficial shave biopsy done for chest lesion and deep punch biopsy for flank lesion both showed poorly differentiated infiltrative carcinoma within the dermis. Immunohistochemistry (IHC) panel showed patchy expression of AE1/AE3 and CK7, with P40 being focally positive, while chromogranin, synaptophysin, CD45, CD3, CD20, S 100, and MelanA were negative.

Patient's family initially opted for further workup in hopes of curative therapy; hence, extensive tumor evaluation was initiated.

The oncology service was consulted to determine the primary tumor. Tumor markers Ca 27.29 and Ca 15.3 (markers for MUCIN gene), Ca 19-9 (marker for pancreatic tumor), CEA (marker for colorectal cancer), AFP (marker for hepatocellular cancer), and PSA (marker for prostatic Ca) were sent, and a bone scan/CT of the whole spine was done as part of the evaluation of an unknown primary and the extent of skeletal metastasis. Both Ca 15.3 and Ca 27.29 were elevated, raising suspicion for a possible metastatic breast cancer.

**Figure 5:**
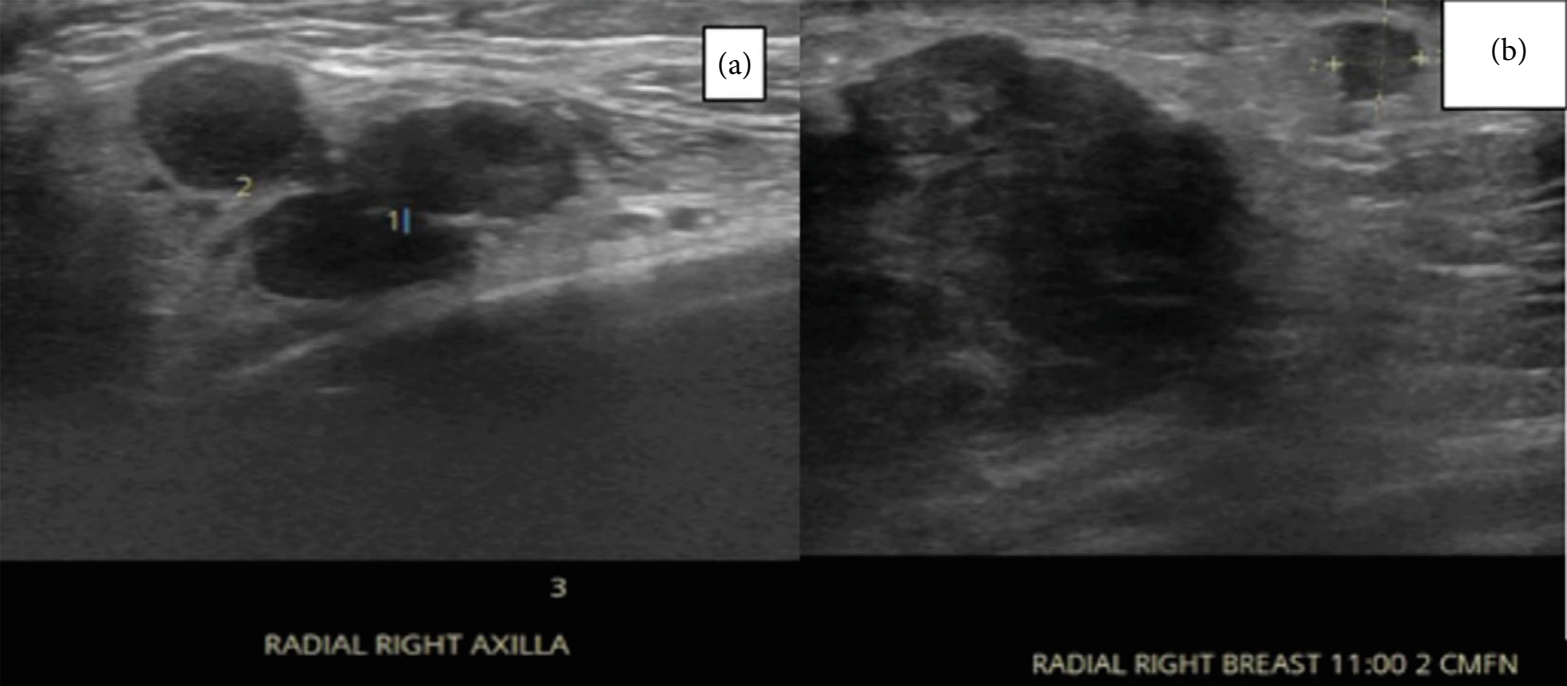
Ultrasound of the right breast and axilla. (a) Targeted breast ultrasound showing a hypoechoic irregular mass with posterior acoustic shadowing, suggestive of malignancy. (b) Axillary ultrasound revealing multiple enlarged, rounded lymph nodes with cortical thickening and loss of fatty hilum, compatible with nodal metastases.

The detection of epithelial markers AE1/AE3, CK7, and P40 on immunochemistry, along with elevated serum tumor markers Ca 15.3 and Ca 27.29, indicated a likelihood of metastatic breast cancer. Consequently, ultrasonography of the breast was performed, revealing multiple microlobular, isoechoic masses, including one external to the dermis, in the upper outer quadrant of the right breast. Enlarged axillary lymph nodes were also observed, raising suspicion for malignancy and metastatic disease with BIRADS 5 classification (Figure 5).

**Figure 6:**
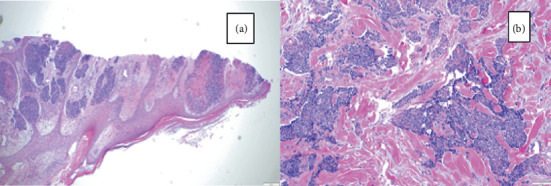
Histopathology of the breast lesion. (a) Hematoxylin and eosin stain showing poorly differentiated invasive ductal carcinoma (Nottingham Grade 3). (b) High-power view demonstrating tubular formation (Score 3), mitotic count (Score 3), and marked nuclear pleomorphism (Score 3) with tumor infiltration into subepithelial connective tissue.

Biopsy of the breast mass revealed poorly differentiated invasive ductal carcinoma. ER, PR, and HER2 receptors were negative (Figure 6). The patient was diagnosed with infiltrative ductal carcinoma of the breast with widespread visceral and skeletal metastases.

After multidisciplinary evaluation and counseling, the family opted for hospice care. Two months later, the patient passed away while under palliative care.

## 3. Discussion

Though previously considered almost identical to its female counterpart, MBC and its distinct genetic and histopathological features have recently been studied by Fox et al. in their 2022 clinical update [5]. They concluded that extrapolating all the literature data on female breast cancer to MBC is problematic. Though they are cancers of the same anatomical site, they have different molecular basis and clinical presentations.

Breast cancer represents less than 1% of all cancers in men. Risk factors include heredity, particularly BRCA2 mutations (found in 26% of cases), reduced testosterone synthesis, and Klinefelter syndrome. MBC usually presents as a well-defined, hard, painless mass behind the areola. Serosanguinous discharge or axillary lymphadenopathy may also occur. Due to its rarity, routine breast cancer screening is not recommended for men. Microcalcifications are uncommon because isolated ductal carcinoma in situ is rare, and screening mammography is not performed in men. MBC is bilateral in 1%–4% of cases, so bilateral mammography is advised even when symptoms are unilateral [7]. MBC typically appears on ultrasound as an irregular hypoechoic cavity with variable attenuation and a peripheral halo. Any cyst detected on ultrasound in men, regardless of characteristics, warrants cytological or histological evaluation. Axillary exploration reveals lymphadenopathy in 60% of breast cancer cases [8]. Breast MRI can be performed in men, although a clearly established consensus indication is lacking. The Breast Imaging Reporting and Data System (BIRADS) MRI criteria are applicable to MBC, as they are to female breast cancer [7].

DCIS is rarely discovered in men, as there is no universal screening mammography for males. Approximately 85% of MBC cases are invasive, with initial diagnoses revealing 37% at Stage 1, 21% at Stage 2, 33% at Stage 3, and 9% at Stage 4 [9]. Most are ductal in origin, and less than 2% of cases are lobular carcinoma. Other common subtypes, like papillary, medullary, and mucinous, were found in 2%, 2%, and 1% of men, respectively. Most MBCs are ER and PR receptor positive and HER2 receptor negative, unlike our patient.

As per the available literature, ER positivity was reported in 80%–99% of cases [10, 11]. Cardoso et al., in a retrospective study of 1483 patients from 1990 to 2010, found that 99% of male patients were ER/PR-positive and HER2-negative, while only 0.3% were triple-negative [12]. The annual protocol of the EORTC 10085/TBCRC/BIG/NABCG International Male Breast Cancer Program reports improved overall and recurrence-free survival in patients with highly ER-positive, PR-positive, and AR-positive disease [12]. The identification of triple-negative status in this case is rare.

Literature review revealed one reported case of triple-negative invasive ductal carcinoma [13]. Li and Qian described a triple-negative MBC with apocrine metaplasia [14].

Serum tumor markers are not recommended for screening [3]. However, the ASCO panel considers it reasonable to assess CA 15-3, CA 27.29, and CEA at baseline in patients with metastatic disease. Serial measurement of these markers can help evaluate treatment response, especially when disease sites cannot be assessed by standard methods. Careful use of serial tumor marker testing may reduce the frequency of radiographic evaluations.

### 3.1. Management of MBC

Due to limited data on MBC, treatment guidelines are typically based on those for female breast cancer. Surgery, chemotherapy, endocrine therapy, and radiation therapy are available treatment options. The choice is determined mainly by the TNM staging, histopathological findings, receptor type, and recurrence score. We will summarize how the treatment strategy in MBC concurs and differs from female breast cancer management.

### 3.2. Surgery

Women with newly diagnosed breast cancer typically receive breast-conserving surgery (BCS), which consists of lumpectomy and whole-breast irradiation. In contrast, most men undergo mastectomy accompanied by either axillary lymph node dissection or sentinel node biopsy. Despite the absence of any medical contraindication, BCS is very uncommon in MBC, even in early stages. Analysis of SEER registry data indicated that only 18% of men with T1N0 tumors underwent BCS. Observation studies have shown that outcomes of BCS are similar to a more radical approach and can be safely used in men, just like in female breast cancer, as BCS offers improved cosmetic and functional outcomes. Sentinel node biopsy is the standard procedure for women with clinically negative lymph nodes. This approach can be extrapolated to men with breast cancer [15].

### 3.3. Radiotherapy

Recommendations of adjuvant radiotherapy in MBC remain similar to female breast cancer. Radiotherapy is often underused in men with breast cancer. SEER data from 1988 to 2012 show that only 42% of men with Stage 1 breast cancer received radiotherapy after BCS. This trend appears similar internationally. In female breast cancer, postoperative radiotherapy is the standard of care after BCS, reducing recurrence by 50% over 10 years and lowering 15-year breast cancer–related mortality by 16% [16]. Postmastectomy irradiation reduces recurrence by over 10% at 10 years in node-positive women and lowers 20-year breast cancer mortality by 8% [16]. Therefore, postmastectomy radiotherapy (PMRT) is routinely administered to female breast cancer patients with positive lymph nodes.

Colciago et al. conducted a meta-analysis to assess the clinical benefit of radiotherapy in metastatic breast cancer. Their pooled analysis of 14 studies reported an aHR of 0.73, indicating an overall 27% reduction in mortality with radiotherapy in MBC, primarily by preventing locoregional recurrences [17]. Hence, RT is recommended as standard of care in MBC just as in female breast cancers [17].

As most of the MBCs are hormone receptor–positive, tamoxifen is recommended following local therapy in early breast cancer or as initial therapy in advanced breast cancer. However, the side effects of tamoxifen may be less tolerable in men. A study showed that in less than 1 year, 21% of men discontinue their treatment because of side effects, compared to just 4% of the women [18]. The management strategies are summarized in ASCO evidence–based treatment guidelines [3]. Of note, these guidelines are mostly designed for management of ER- and PR-positive MBCs and would be hardly applicable in our case had curative treatment been chosen.

### 3.4. Skin Involvement in MBC

In contrast to female breast cancer, MBC in addition to the breast lump, presents with skin lesions involving the areola, nipple–areolar complex, or the entire skin. In some instances, cutaneous manifestations may serve as the primary presenting feature, as in our case. Skin metastasis in breast cancer can be synchronous or metachronous. Ratón et al. studied the skin manifestations of MBC over the span of 5 years and noted the following findings. Male breast carcinoma generally has a less favorable prognosis than female breast cancer, influenced by anatomical factors, clinical stage, histopathologic type, and patient age. The overall 5-year survival rate ranges from 22% to 72% across all stages. Skin changes typically do not affect prognosis, except in cases of Paget disease, which is linked to poorer outcomes in men [19]. While most case reports discussed the skin involvement of the MBC in part of the skin adjacent to nipple–areolar complex, only a few cases report distant skin metastasis. Jessica Kim et al. report a case of MBC with skin metastasis to the groin and scrotum, highlighting the potential extent of skin involvement in this disease [8]. MBC with skin metastasis is rare, with only four reported cases. Of these, just two describe exophytic areolar lesions as the initial presentation [20, 21]. A review of the literature indicates that cutaneous metastases (CMs) account for approximately 2% of all cutaneous tumors [22]. So CMs are much less prevalent compared to the primary skin malignancy. Most available data on the CMs from breast cancer is derived from studies on female patients. With that said, it was shown that the frequency of CMs is 1%–10% among all metastatic cancers, with higher CM noted in breast cancer (18%–26% of all cases) [22, 23]. At the same time, CMs originating from the breast were diagnosed before the primary tumor only in 3% of cases of breast cancer, which is significantly less often than in the case of lung, stomach, ovary, and kidney cancers [24]. Our patient is among the rare cases where CMs were identified before the primary tumor. Metastatic skin lesions from breast cancer are typically nodular and occur on the same side as the primary tumor, though other forms, such as multiple telangiectatic papules, peau d'orange, and carcinoma en cuirasse, have been reported. In our case, the CMs appeared ipsilaterally across two body regions (axilla and the flank).

A skin biopsy is essential for distinguishing between primary skin tumors and CMs. Histological characteristics differ based on the primary malignancy and aid in identifying the occult primary. When relevant clinical history is lacking, determining the primary site of metastatic cancers can be challenging. Metastases typically exhibit histopathological features similar to those of the primary tumor. Nevertheless, in approximately 5%–10% of cases, the primary site remains unidentified [24]. IHC in breast cancer typically shows a CK7-positive and CK20-negative cytokeratin pattern. ER and PR further improve detection sensitivity. In this case, the initial skin mass biopsy revealed a poorly differentiated carcinoma with patchy AE1/AE3, CK7, and P40 positivity on IHC. Only elevated serum tumor markers Ca 15.3 and Ca 27.29 indicated the lesion's breast origin.

### 3.5. Prognosis

As per a large French cohort of 489 patients from 1990 to 2005, early diagnosis and use of adjuvant Endocrine therapy (ET)/Chemotherapy (CT)/Radiotherapy (RT) were associated with lower disease recurrence and increased survival rates. Prognostic factors were similar to women, including tumor size and nodal status. Other poor prognostic factors are older age at diagnosis, triple-negative disease, and HER2 positivity [25].

Analysis of outcome data from 322 men in the SEER registry who underwent recurrence scoring with genomic tests such as Oncotype DX and MammoPrint demonstrates the prognostic value of these assessments in male patients. Five-year breast cancer–specific survival rates among men were 99.0% for those with a recurrence score below 18, 95.9% for scores between 18 and 30, and 81.0% for scores of 31 or higher. In comparison, the corresponding survival rates among women were 99.5%, 98.6%, and 94.9%. Despite later onset and more advanced disease at diagnosis, MBC patients show better survival than female breast cancer patients. After adjusting for age, cancer stage, and treatment, the relative excess risk is 0.78, according to a multinational population-based study [6].

An analysis of the SEER cancer database by Auvinen found that men with a history of breast cancer have a 30-fold increased risk of developing contralateral breast cancer. The absolute risk of a second primary breast cancer in men is estimated at approximately 1.75% [26]. This population is also associated with an increased risk of developing melanoma and prostate cancer [26].

## 4. Future Studies

Although male patients have historically been understudied, a limited number of clinical trials with inclusive recruitment are currently in progress. One Japanese Phase II study is evaluating the safety and efficacy of the phosphatidylinositol-4,5-bisphosphate 3-kinase catalytic subunit alpha (PIK3CA) inhibitor, alpelisib, in combination with fulvestrant in men and postmenopausal women with ER/PR-positive, HER2-negative, advanced breast cancer harboring a PIK3CA mutation. Eligible participants have experienced disease progression on or after aromatase inhibitor treatment, regardless of prior CDK4/6 inhibitor use. Another ongoing study is analyzing a large series of metastatic breast cancer tumor specimens to determine their biological characteristics and to identify relevant prognostic and predictive markers.

## 5. Conclusion

MBC is increasingly common owing to the demographic shift. Our patient with the initial presentation as a skin tumor away from the nipple–areolar complex is a clinical oddity. Our case report is intended to shine light on this relatively rarer presentation of a rare form of tumor.

## Data Availability

Data sharing is not applicable to this article as no datasets were generated or analyzed during the current study.
